# Incidental Diagnosis of a Rare Case of Corkscrew Aorta

**DOI:** 10.1155/2019/2016959

**Published:** 2019-10-13

**Authors:** M. Petullà, G. Mazzarella, L. Critelli, L. Paone, D. Laganà

**Affiliations:** Division of Radiology, Department of Clinical and Experimental Medicine, “Magna Græcia” University, Catanzaro, Italy

## Abstract

The corkscrew aorta is a variant of the normal anatomical course of the aorta. This rare condition is characterized by a marked tortuosity of the aorta. In our experience it concerns the tract of subrenal aorta, that is an unusual condition, since there are no other cases in the literature. It is characterized by the presence of at least two kinking, and a coiling interposed among them. It is diagnosed by Angio-CT and its response is incidental, being from an asymptomatic clinical point of view.

## 1. Introduction

The “corkscrew aorta” is a variant of the normal anatomical course of the aorta. This is a rare condition of which there are no cases in the literature in reference to the abdominal aorta, having been described only in the cervical and thoracic district, mostly in the pediatric age. In particular, it was found tortuous course of the aortic arch with corkscrew appearance, sometimes in association with other vascular anomalies [1, 2]. The symptomatology, in these cases, was mainly characterized by syncopal episodes since a situation similar to aortic coarctation was determined. In our case, the variant “corkscrew aorta” involved the abdominal subrenal tract. Proceeding downward from the top, the abdominal aorta is usually present in a straight line and progressively decreases its caliber. Sometimes it may present curves or undulations such as to give it the terms of a sinuous aorta, while in our case it assumed a clear corkscrew appearance. From the clinical point of view, although its finding occurs incidentally, it could determine hypoinflux to the lower limbs, due to possible strictures in the affected tract.

## 2. Case Report

A 79-year-old man, with a history of HCV-related cirrhosis, arrives in our operative unit to perform an abdominal ultrasound in which biliary tract dilatation is observed.

**Figure 1:**
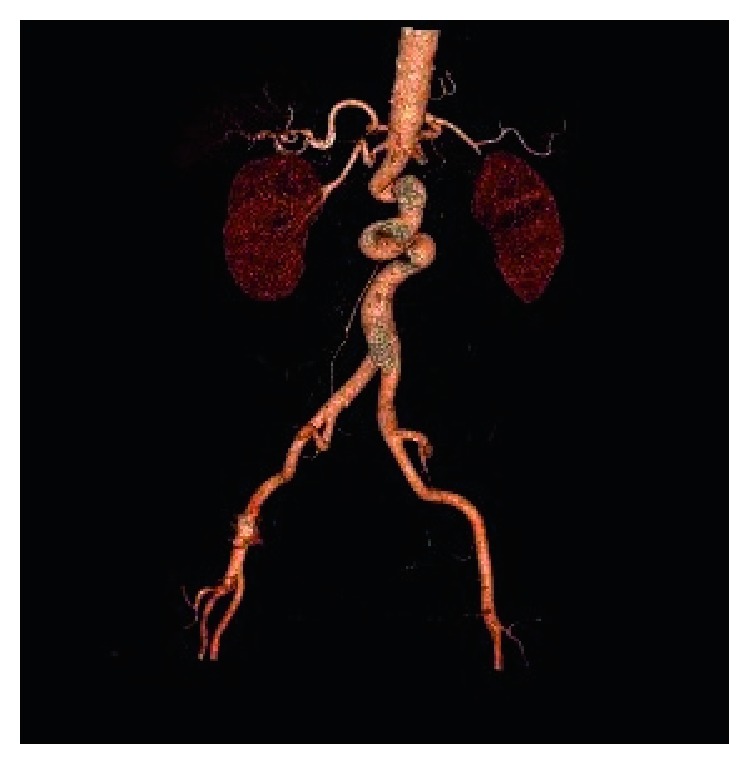
3D recostruction of the abdominal aorta demonstrates the presence of at least two kinking and a coiling.

**Figure 2:**
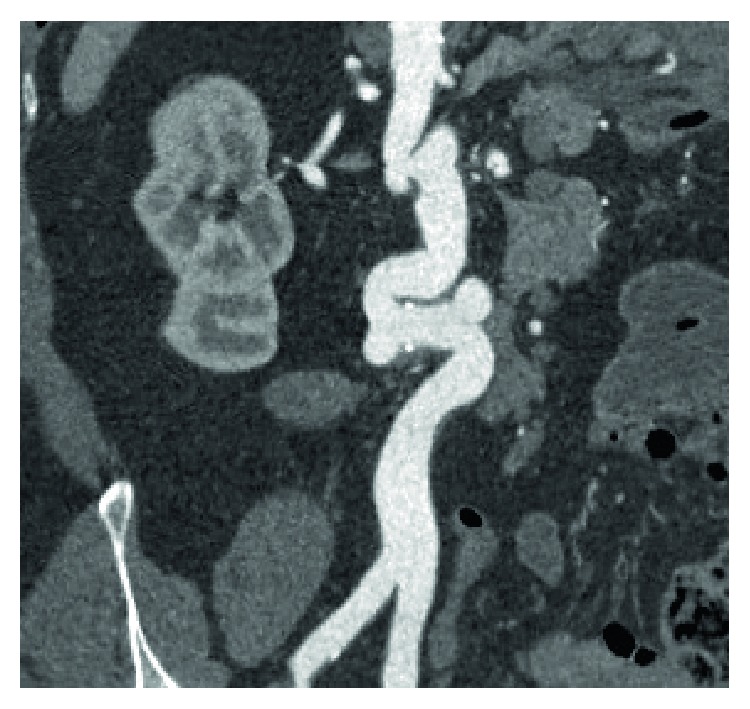
MPR recostruction of the abdominal aorta.

For this reason we make a Colangio-RMN (Achieva, Philips 1.5T) with T Ip/Op, T2 Ssh, SPAIR, DWIBS and images acquired according to the axial and coronal planes, integrated with MRCP 2D, 3D, where the abnormality of the abdominal aorta was accidentally detected. Therefore, to evaluate the presence of further abnormalities of the thoracoabdominal aorta, we make an Angio-CT (Aquilion, Toshiba 64 layers) with contrast medium (iomeprol 400: volume administered: 90 ml: infusion speed: 4.5 ml/sec), with three dimensional and multi-planar reconstructions in postprocessing (Figures 1 and 2).

The aorta showed dimensions within the limits of the norm with the presence of parietal calcifications, and typically tortuous course only in its subrenal tract, in fact no variation of the arch, thoracic aorta, supraaortic trunks, and visceral vessels were found. In the effected subrenal tract (at 2.8 cm from the inferior renal artery) two kinking and coiling interposed among them were appreciated, without any significant stenosis or pathological dilation. The emergence of downstream vessels, the superior and inferior mesenteric artery, was typically without variations.

The aorta regained regular course at the bifurcation with regular iliac axes by size and course.

The patient was then subjected to clinical and instrumental supervision with an ultrasound examination on the 3rd and 6th months and Angio CT on the 12th month to evaluate any changes or appearance of clinical signs.

## 3. Discussion

The corkscrew aorta is a variant of the normal anatomical course of the aorta. This is a rare condition of which there are no cases in the literature referring to the abdominal aorta, having been found in the cervical and thoracic district [1, 2]. It is characterized by a marked tortuosity of the aorta in its subrenal tract.

The diagnosis is incidental, carried out exclusively with diagnostic images, particularly with Angio-CT. This demonstrates the presence of at least two kinking [3, 4], defined as elongation that cause the formation of an angle of about 90° and a coiling defined instead as an elongation rounding, with tightening on the longitudinal axis (Figures 1 and 2).

In our experience this condition did not determine any symptoms [5, 6].

Among the complications cannot be excluded are the formation of stenosis or narrowing, due to the presence of multiple angles, as it happens on other vascular districts, which could determine hypoinflux to the lower limbs, but in our work this possibility did not occur since the morphological picture remained unchanged and iconographic after a year. If complications had occurred, the only treatment available would have been aorto-aortic grafting, since the tortuosity of the vessel contraindicates endovascular treatment.

## 4. Conclusion

The corkscrew aorta, defined as a variant of the normal anatomical course of the aorta, is incidental, confirmed by Angio-CT, being from an asymptomatic clinical point of view.
